# Spontaneous Pregnancy After Isotretinoin Use in a Man After Unsuccessful Microdissection Testicular Sperm Extraction

**DOI:** 10.7759/cureus.96813

**Published:** 2025-11-14

**Authors:** Zaed Jaber, Kerat Hanspal, Osama Jaber, Ranjith Ramasamy

**Affiliations:** 1 Medicine, Mohammed Bin Rashid University of Medicine and Health Sciences, Dubai, ARE; 2 Urology, Uro Diagnostic Clinic, Dubai, ARE; 3 Urology, Jumeirah American Clinic, Dubai, ARE

**Keywords:** – isotretinoin, microdissection testicular sperm extraction, nonobstructive azoospermia, pregnancy, spermatogenesis, spontaneous conception

## Abstract

We report a spontaneous pregnancy in the partner of a man with non-obstructive azoospermia and histologically confirmed late maturation arrest following a failed microdissection testicular sperm extraction (micro-TESE). This patient was subsequently treated with isotretinoin for six months and, thereafter, conceived naturally without any assisted reproductive techniques. This case suggests the possibility of improvement in sperm production in men with nonobstructive azoospermia (NOA) despite lack of success with surgical sperm retrieval and implicates isotretinoin as a potential therapeutic agent that is noninvasive and cost-effective. While causality cannot be established from a single case, these findings warrant further investigation in prospective studies.

## Introduction

Nonobstructive azoospermia (NOA) is a severe type of male infertility in which the testes are unable to produce sperm [1], often secondary to endogenous defects in spermatogenesis, and NOA is one histopathological expression of maturation arrest, where the germ cells are present but fail to differentiate past a specific developmental phase and thus sperm are absent in the ejaculate.

Our case is unique in that, following an unsuccessful microdissection testicular sperm extraction (micro-TESE), a spontaneous pregnancy developed in the partner of a man with NOA and histologically confirmed maturation arrest. Early-stage germ cells are found in the seminiferous tubules in NOA due to maturation arrest, but they are unable to differentiate into mature spermatozoa. As a result, there are no sperm in the ejaculate, and surgical retrieval is frequently unsuccessful [2,3]. However, in this instance, the patient received postoperative treatment with isotretinoin, a retinoid that is known to alter retinoic acid (RA)-dependent pathways that are essential for meiotic entry and spermatogonial differentiation [4,5]. Following treatment, the couple achieved a natural conception without the use of assisted reproductive technologies. To our knowledge, this is the first documented report of a spontaneous pregnancy occurring after failed micro-TESE in a man with maturation arrest who subsequently received isotretinoin therapy.

Isotretinoin is a systemic retinoid (13-cis retinoic acid), a derivative of vitamin A that is closely related to RA, the active form responsible for regulating spermatogenesis. RA plays a vital role in normal spermatogenesis by controlling spermatogonia differentiation, entry into meiosis, germ-Sertoli cell contact, and the initiation of spermatogenesis [4,5]. Testicular RA levels have been shown to correlate with sperm production in infertile men, highlighting their clinical relevance. Systemic isotretinoin, commonly used in dermatology, has been suggested as a treatment to restore failed spermatogenic pathways by modulating the RA signaling in testicular tissue [6]. Although its teratogenic effects on the reproductive age of a woman are well-known and highly regulated [7], its impact on male fertility has not been studied as broadly. Multiple human trials in men, in both acne treatment scenarios and infertility settings, have shown an improvement in semen parameters, such as sperm concentration, motility, and vitality, with variable effects on sperm morphology [6,8,9]. These findings raise the possibility that isotretinoin could have a therapeutic role in certain forms of male infertility, though clinical evidence remains limited.

## Case presentation

**Figure 1:**
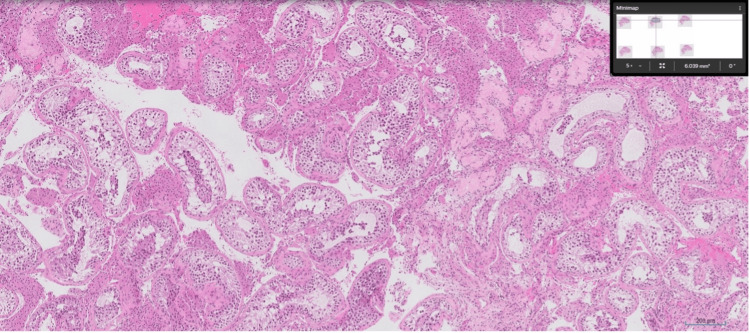
Hematoxylin and eosin (H&E)-stained section of testicular tissue showing seminiferous tubules with germ cell maturation arrest. Early spermatogenic cells are present, but spermatids and spermatozoa are absent

**Figure 2:**
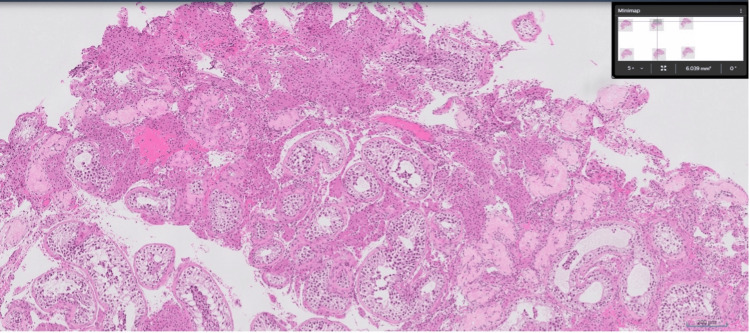
Another field from the same biopsy specimen demonstrates similar findings of maturation arrest, with preservation of Sertoli and Leydig cells

The chronology of events in our case is unique because the patient, a 32-year-old, had previously undergone micro-TESE, with microscopic documentation showing that no sperm were obtained, which was further supported by a sperm analysis showing zero sperm in a 2 mL semen sample. Testicular biopsy histological analysis revealed complete maturation arrest in 50-70% of the tubules (Figures 1, 2). However, after receiving isotretinoin therapy 10 mg once a day for six months, his 31-year-old partner experienced a spontaneous pregnancy, indicating that isotretinoin might have activated spermatogenic potential that was not detected during the initial surgical retrieval. Semen analysis at six months after starting isotretinoin showed a volume of 2 mL, sperm concentration of 1 million/mL, and 30% motility. A repeat semen analysis at nine months showed a volume of 2.5 mL, sperm concentration of 1.5 million/mL, and 38% motility. The patient also experienced mild side effects, including lip chapping, which he managed with regular use of moisturizing cream. Importantly, in our patient’s partner, now at 16 weeks’ gestation, ultrasound showed normal development with no congenital abnormalities, suggesting that indirect exposure via paternal therapy did not result in teratogenic effects.

## Discussion

There is a small but increasing body of evidence of the beneficial effects of isotretinoin on spermatogenesis. Amory et al. conducted a study using isotretinoin to treat men with NOA over a 20-week period and found that a small portion of patients demonstrated recovery of sperm in their semen analysis after treatment [8,10]. More recently, Gurel et al. studied 81 men with acne treated with isotretinoin (median dose 120 mg/kg cumulative). They found that sperm concentration, motility, vitality, and total count significantly improved, but morphology declined [9]. In men with NOA or cryptozoospermia, a recent prospective trial assessed isotretinoin 20 mg twice daily for three to nine months, offering the first comprehensive proof of its possible function [11]. In that study, 37% of treated men developed reliable, motile ejaculated sperm, sometimes sufficient for in vitro fertilization (IVF)/intracytoplasmic sperm injection (ICSI) without surgical retrieval. Interestingly, the largest subgroup of responders was men with maturation arrest histology, which matched the histological profile of our patient. Similar to our case, some of the men in the prospective trial responded to isotretinoin even though the majority had previously failed retrieval attempts. Dry skin, irritability, and occasional lipid changes were among the frequent but mild side effects, indicating acceptable tolerability [11].

In NOA, micro-TESE is thought to be the most sensitive technique for sperm retrieval, and a failed attempt is usually regarded as a clear sign of a poor prognosis. Micro-TESE success in patients with diffuse maturation arrest is reported to be very low (~35%), as the uniform tubular architecture limits the possibility of identifying focal areas of active spermatogenesis [12]. Due to the focal and patchy nature of spermatogenesis in NOA, limited sampling may miss uncommon foci of sperm production, despite the high sensitivity of micro-TESE [13-15]. The temporal association suggests that isotretinoin may have modulated residual spermatogenic activity, even though causality cannot be established from a single case. It is noteworthy that rare spontaneous recovery of fertility in NOA has been documented in other contexts [16].

This report's main strength is its novelty: to the best of our knowledge, this is the first instance of spontaneous pregnancy following a failed micro-TESE in a man who had histologically confirmed maturation arrest (Figures 1, 2) and was given isotretinoin. Mechanistic and early clinical evidence support the case, which is biologically plausible and suggests that retinoid therapy may be useful in treating severe male infertility. Limitations include the inability to prove causality, the rare possibility of spontaneous spermatogenic recovery, the absence of detailed semen analyses before or after therapy, and the lack of long-term follow-up, all of which limit the generalizability of these findings.

These surgeries are invasive and costly, and studies indicate micro-TESE retrieved sperm in ~46.6% of men with NOA (range: 18-71%) [17]. In our case, the use of isotretinoin may be a low-cost and noninvasive complementary treatment or alternative when standalone treatment options have failed, possibly minimizing the number of surgical procedures and attempts. However, the drop in morphology reported by Gurel et al. causes concern about sperm quality and drives the urgency that additional trials should be conducted [9]. Furthermore, preoperative testicular biopsy demonstrating maturation arrest may help identify candidates for isotretinoin therapy before proceeding with micro-TESE, potentially reducing unnecessary surgical interventions. Importantly, paternal exposure has not been demonstrated to present a teratogenic risk, which is consistent with our case outcome, even though isotretinoin's teratogenicity in women of reproductive age is well-established [18,19]. Men who take the medication can detect isotretinoin in their semen, but the amount is incredibly small, roughly a million times less than what is found in a typical oral dosage of 40 mg. This trace amount is regarded as insignificant and is not anticipated to present a teratogenic risk to a prospective fetus or partner. Pregnancy prevention measures are only required for patients who have the potential to become pregnant, according to the American Academy of Dermatology's 2024 guidelines. Male patients taking isotretinoin who are trying to conceive are exempt from the need for contraception or abstinence [20].

Future directions

This case demonstrates the importance of conducting larger-scale and prospective studies that will examine isotretinoin as a beneficial therapeutic strategy in NOA. The main unanswered questions are the optimum dose to use, the duration of therapy, and the long-term safety of the therapy, as well as the nature of patients who would be likely to benefit. Moreover, molecular indicators of RA deficiency within the testes can be used to profile responders versus nonresponders.

Key Considerations From This Case

Unexpected recovery potential: Even in the context of failed micro-TESE with no sperm histologically identified, spermatogenesis may still be inducible.

Therapeutic implications: Isotretinoin could serve as a noninvasive adjunct therapy in NOA, especially in maturation arrest.

Diagnostic caution: The failure of the micro-TESE may not mean that medical intervention to stimulate sperm production should be excluded.

Need for evidence: Randomized controlled trials are warranted before isotretinoin can be incorporated into clinical practice for male infertility.

## Conclusions

In this case, a man with histologically confirmed maturation arrest who later received isotretinoin therapy experienced a rare but notable case of spontaneous pregnancy after failing micro-TESE. Isotretinoin may play a part in regulating spermatogenesis in NOA, according to the temporal sequence and supporting mechanistic evidence. These results imply that by altering RA pathways, isotretinoin may promote spermatogenesis, especially in patients experiencing maturation arrest. These findings call for more clinical research on retinoid-based treatments as a potential cure for male infertility.
